# Detection of *Coccidioides posadasii* from xerophytic environments in Venezuela reveals risk of naturally acquired coccidioidomycosis infections

**DOI:** 10.1038/s41426-018-0049-6

**Published:** 2018-03-29

**Authors:** Primavera Alvarado, Marcus de Melo Teixeira, Lela Andrews, Alexis Fernandez, Gerardo Santander, Adina Doyle, Magaly Perez, Francisco Yegres, Bridget Marie Barker

**Affiliations:** 1 Laboratorio de Micología, Servicio Autonomo Instituto de Biomedicina Dr. Jacinto Convit, Caracas, 4043 Venezuela; 2 Pathogen and Microbiome Institute, Northern Arizona University, Flagstaff, AZ 86011 USA; 3 Environmental Genetics and Genomics Laboratory, Northern Arizona University, Flagstaff, AZ 86011 USA; 4 Laboratorio de Inmunología II, Servicio Autónomo Instituto de Biomedicina Dr. Jacinto Convit, Caracas, 4043 Venezuela; 5 Laboratory Geomatics, Universidad Bolivariana de Venezuela, Caracas, 1040 Venezuela; 6 Division of Pathogen Genomics, Translational Genomics Research Institute-North, Flagstaff, AZ 86005 USA; 7 Laboratorio de Investigación y Apoyo Docente del Santa Ana (LIADSA), Universidad Nacional Experimental Francisco de Miranda (UNEFM), Coro, 4101 Venezuela

## Abstract

A wide range of mammals are susceptible to infection by the fungal species *Coccidioides immitis* and *C. posadasii*. In humans, 60% of infections are asymptomatic; however, certain patients may develop a severe and deep systemic mycosis called coccidioidomycosis. Genetic analysis suggests that the majority of clinical isolates recovered from South America are *C. posadasii*; however, little is known about the prevalence, species distribution, and ecological factors that favor the occurrence of this pathogen in those areas. By using a combined quantitative polymerase chain reaction (qPCR)-based approach and mycobiome amplicon sequencing, we provide evidence that at least two genotypes of *C. posadasii* are found in the xerophytic environment in Venezuela. We detected a 3806-fold range in the amount of *Coccidioides* DNA when comparing among the sampled locations, which indicates that human exposure risk is variable, and is one critical factor for disease manifestation. We identified fungal communities that are correlated with a higher prevalence of *C. posadasii*, suggesting that a combination of specific microbes and a xeric microenvironment may favor the growth of *Coccidioides* in certain locations. Moreover, we discuss the use of a combinatorial approach, using both qPCR and deep-sequencing methods to assess and monitor fungal pathogen burden at outbreak sources.

## Introduction

Coccidioidomycosis can be a severe systemic mycosis caused by two dimorphic fungal species *Coccidioides immitis* and *C. posadasii*^1^. The area of highest disease incidence overlaps with arid and semi-arid regions of both American continents, ranging from the western United States to northern Argentina^2–5^. These areas are characterized by high salinity soils and xeric conditions that support specific biological communities with ecological and physiological adaptations for survival in these extreme environments^6^. When growing in the environment, it is thought that *Coccidioides* spp. are saprobes, and associate with environments enriched in animal-derived material and high average annual temperatures^1, 7–11^. Both species of *Coccidioides* grow as mycelia in the soil, and produce air-borne arthroconidia that can be inhaled by susceptible hosts, which initiates infection^2, 3^. In the body, the fungus undergoes a morphological shift to endosporulating spherules that can be cleared, go quiescent, or progress into a pulmonary or disseminated mycosis. Beyond humans, *Coccidioides* spp. have been isolated from bats, desert rodents, armadillos, dogs, and many other mammalian species in North and South America^12–19^.

Population genetics studies suggest at least three populations within *C. immitis*: San Joaquin/Central Valley California, Southern California/Mexico and Washington^1, 20–23^. Genetic analysis of *C. posadasii* proposes four main populations: Texas/South America, Mexico, Guatemala, and Arizona^1, 20, 21, 23^. Recently, an even more refined population structure within Arizona was proposed. Yuma and Phoenix clinical isolates were shown to be genetically distinct from those obtained from patients in Tucson. A third Arizona sub-population containing primarily soil and veterinary isolates was identified, which may reflect differences in ecological adaptation, host specificity, and/or virulence^21, 23^.

*Coccidioides immitis, C. posadasii*, and other related Onygenales species can colonize humans and other mammalian species, possibly because these organisms possess metabolic pathways that can degrade animal-derived compounds, are thermotolerant, and often dimorphic^24–27^. Factors associated with presence and distribution of *Coccidioides* in the environment have been examined, with the goal of understanding fungal eco-epidemiology^7, 8, 11, 22, 28–31^. Early studies have shown that *Coccidioides* can be isolated from soil, albeit with low success rate, via inoculation in susceptible mice, or by seeding contaminated soil on selective agar^32–34^. Studies using extensive soil mapping indicated a sporadic and localized distribution of the fungus^10, 35^.

Advanced molecular biology techniques used to detect the fungus in the environment have been developed, and have helped to understand and define the environmental niche of *Coccidioides*^36^. Various PCR-based methods for the detection of *Coccidioides* DNA from soil and dust have become a more common tool for environmental detection of these fungi, and have the promise to increase efficiency when compared to direct plating or mouse passage^22, 28, 37–39^. However, metagenomic sequencing data to identify operational taxonomic units (OTUs)^40^ that represent the *Coccidioides* genus in both clinical and environmental contexts are still needed.

Overall, there is a paucity of data describing the prevalence and genetic profiling of *Coccidioides* in South America. The majority of ecological studies of *C. posadasii* have been conducted in the Sonoran desert of the American state of Arizona and Northern Mexico, which is geographically distant and isolated from other arid/semi-arid regions of Brazil, Argentina, Paraguay, Colombia, and Venezuela in which *C. posadasii* is also endemic^41^. The few clinical and environmental samples described thus far from South America group within the *C. posadasii* Texas/Mexico population; however, a better understanding regarding *Coccidioides’* prevalence and the associated soil fungal community for endemic sites within South America is still needed^20, 21^.

Geographically, Venezuela is an important link between North, Central, and South America. The nearest confirmed endemic area in Central America is Guatemala, where a single *C. posadasii* population has been described, which is genetically distinct from South American isolates from Brazil and Paraguay^21^. Coccidioidomycosis cases have been reported in Venezuela since 1948, primarily in the semi-desert northwestern regions of the country between 10° and 12° north latitude^41, 42^. The arid and semi-arid endemic zone is restricted to Zulia, Lara, and Falcón states, which are characterized by warm, arid and dry climates, low altitude, xerophytic vegetation, sandy soils with high-salt concentrations (boron and calcium sulfate) and alkaline pH, which is presumably favorable for *Coccidioides* development^41, 43^. Local rural populations and travelers to these areas of Venezuela are at risk for acquiring the disease^44, 45^. To date, 114 cases of coccidioidomycosis have been reported in Venezuela with the majority from the northwestern area of Falcón state. We propose that this is a fraction of actual cases, which are likely under-reported or misdiagnosed due to a lack of awareness of the disease among health care providers and the public^42, 46–48^. *Coccidioides* was reported to have been isolated from soil samples from Paraguaná peninsula, located in the endemic Falcón state. However, the genetic profile of this soil-derived isolate was not verified and subsequent infections in rats did not show any formation of spherules as observed in biopsies of patients with coccidioidomycosis in Venezuela^43^.

This study aimed to: (1) Use molecular detection of *Coccidioides* spp. in soil samples in endemic areas of Venezuela; (2) Compare the genetic profile of soil-derived *Coccidioides* ITS2 PCR amplicons generated by high-throughput sequencing with clinical-derived sequences available in the GENBANK; and (3) Compare the mycobiome composition between low-positive and high-positive sites to identify fungal communities that may be associated high prevalence of *Coccidioides*.

## Materials and methods

### Soil collection and DNA extraction

Fifteen soil samples were collected in the municipalities of Urumaco, Sucre, and Democracia in Falcon state as well in Urdaneta and Torres located in the Lara state (Table 1). These sites represent variation in soil type, and physiographic position on the landscape. Samples were collected between April and July, which represents the dry seasons in Venezuela. Detailed descriptions of the soil and vegetation-type, collection dates, and Global Positioning System (GPS) readings for each site are reported in Table 1.

**Table 1:** Site identification, location, sampling data, temperature range, rain precipitation, geographic coordinates, soil type, and vegetation-type of samples areas in Lara and Falcon states of Venezuela

Sites	Location	Date of collection	Mean temperature range	Annual precipitation (mm)	Geographic coordinates	Soil type	Vegetation
1 and 2	Urumaco (San José de Bruzual)	04/13/2015	>34 °C	600–800	70°19'48,328"W 11°4'47,769"N	Clay	Shrubs tropophilous deciduous and semi-deciduous
3, 4, and 5	Sucre (Pedregal)	04/15/2015	32–34 °C	500–600	69°51'49,916"W 11°4'27,331"N	Clay	Tropophilous forest basimontane
6, 7, and 8	Democracia (Pecaya)	04/21/2015	32–34 °C	400–500	70°7'13,178"W 11°1'17,091"N	French-sandy	Spiny xerophytic bushes
9, 10, and 11	Urdaneta (Siquisique)	07/20/2015	32–34 °C	<400	69°45'8344"W 10°35'4025"N	Clay-sandy	Agricultural soil
12, 13, and 14	Torres (La Majada)	07/22/2015	30–32 °C	600–800	70°10'14,917"W 10°17'41,557"N	Clay	Thorny xeric shrublands
15	Torres (La Burra)	07/23/2015	28–30 °C	800–1000	70°25'56,35"W 9°58'54,79"N	Clay	Shrubs tropophilous deciduous and semi-deciduous

Soil samples were collected at random in each of the locations with a hand shovel, 10 cm wide and 20 cm deep. Soil samples were air-dried and stored at room temperature. Four DNA extractions from a single soil sample were performed from each of the 15 areas using the PowerSoil® DNA Isolation Kit (MO BIO Laboratories). Approximately 0.25 g of soil was added to each PowerBead tube, and DNA extraction was performed according to the manufacturer’s instructions. DNA samples were further purified with PEG-8000 and carboxylated magnetic beads^49^ quantified by PicoGreen (Life Technologies, Inc, Carlsbad, CA) fluorescence, and normalized to a final concentration of 10 ng/µL.

### Soil culturing and inoculation in mice

The 15 soil samples herein evaluated from the Lara and Falcón states were processed for both direct plating on selective agar and inoculation in mice in order to isolate *Coccidioides* sp. The soil suspensions were prepared following the protocol described by Barker et al.^31^ with some modifications. Briefly, 5 g of each collected soil sample was placed in 50 mL conical tubes. Subsequently, the soil was hydrated with 25 mL of 30% NaCl and 0.01% Tween 80. The tubes were shaken until the soil particles were disintegrated and allowed to settle for 30 min. Ten milliliters of the supernatant was collected and transferred into new 50 ml conical tubes. Forty milliliters of sterile distilled water was added and centrifuged at 3000 × *g* for 40 min at 4 °C. The pellet was re-suspended in 25 mL of sterile water and centrifuged again at 3000 ×* g* for 40 min at 4 °C. Finally, the sediment of one tube was re-suspended with 2 mL of distilled water or with 0.9% saline solution supplemented with streptomycin (30 μg/mL). Two-hundred fifty microliters of the volume was seeded onto four Mycosel agar Petri dishes. Two plates were incubated at 24 °C and two plates were incubated at 37 °C. The presence of *Coccidioides*-like mycelial colonies was evaluated up to 4 weeks.

Inoculation in mice was conducted under the approval of the Bioethics Committee of the Autonomous Institute of Biomedicine Dr. Jacinto Convit. Sixty BALB/c male mice (6–8 weeks of age) were inoculated intraperitoneally with 0.5 mL of soil suspension in 0.9% saline solution and streptomycin (30 μg/mL—see above). Four mice were inoculated for each processed sample and followed daily until euthanasia. Mice inoculated with sterile saline were used as negative controls. Positive control mice were inoculated with a *Coccidioides posadasii* strain at a concentration of McFarland 7. After 30 days of inoculation, the mice were sacrificed and any evidence of the disease was recorded. Samples from lungs, liver and spleen were collected, processed, and seeded in Sabouraud agar medium with 150 mg/L of chloramphenicol. The cultures were incubated at 24 °C for and observed for *Coccidioides* sp. grow for up to 4 weeks. Animals that died before 30 days were immediately processed.

### Molecular detection of *Coccidioides* spp. by qPCR

A real-time qPCR-based assay was employed to screen for *Coccidioides* in the Venezuelan soil samples, which is derived from an FDA approved clinical assay (https://www.accessdata.fda.gov/scripts/cdrh/cfdocs/cfpmn/denovo.cfm?ID = DEN170041)^50–53^. The assay was performed on the Applied Biosystems 7900HT Real-Time PCR System (Thermo Fisher Scientific) and each 10 µL reaction mixture contained 1 × PerfeCT Taq PCR Fast Mix II, ROX background dye (Quanta Biosciences, Gaithersburg, MD), 1 × Cocci Assay mix (detailed in Table 2) and 20 ng DNA template^50^. Cycling conditions were initial denaturation for 10 min at 95 °C, followed by 40 cycles of 15 s at 95 °C and 1 min at 60 °C. Real-time PCR assays were performed in triplicate. Control samples included purified DNA from Silveira strain of *C. posadasii* as a positive control, and no-template controls included water and previously known negative soils for *Coccidioides* spp. recovered from Flagstaff, Arizona. A soil was considered “high positive” if *C*_t_ value were ≤35 and all soil DNA extractions amplified, and a “moderate positive” if *C*_t_ >35. Low/not positive soils were considered those when three or fewer of the four soil DNA extractions did not show detectable real-time PCR results.

**Table 2:** CocciEnv primers and probe

CocciEnv assay primers	Name	Sequence 3′–5′	Final conc. (μM)
Forward	CocciEnv_F1d1	CGTTGCACRGGGAGCACCT	0.375
	CocciEnv_F2	AAGCTTTGGATCTTTGTGGCTCT	0.375
	CocciEnv_F3	AATTGATCCATTGCAAGCACCT	0.25
	CocciEnv_F4	AATCCAACCTTTGGAACTACACCT	0.25
	CocciEnv_F5	TTTTCCGGTATGGACTAGCACCT	0.375
	CocciEnv_F6d2	TGTTAGGTAATCYAACYAGCACCT	0.125
	CocciEnv_F7d2	TRTTAGGTAATYCAACTAGCACCT	0.125
	CocciEnv_F8d1	TGTTAGATAATCCAACYAGCACCT	0.125
	CocciEnv_F9d2	GKTARGTAATCCAACTAGCACCT	0.125
	CocciEnv_F10d2	TGTTAGGTARTCCAACTAGCAYCT	0.125
	CocciEnv_F11d2	TGTTAGGTAATCCAACTMGCACYT	0.125
Reverse	CocciEnv_R1	GATGGAGGACTCTATATGCTTGT	0.375
	CocciEnv_R2	ATGGAGGACTCGTTATGCCTGT	0.375
	CocciEnv_R3	GGAGGACCCGTATGCTTGTGT	0.375
	CocciEnv_R4	TGCTAAATGATGGAGGGCTTGT	0.375
	CocciEnv_R5	GATGGAGGCTCGTATGCTTGT	0.375
	CocciEnv_R6	AAGGGGTTTGTGGTGAATCCTTA	0.375
	CocciEnv_R7	CAGAAAAATAGCCGTATGCTTGT	0.375
	CocciEnv_R8d2	TRATGGAGRACTTGTATGCTTGT	0.125
	CocciEnv_R9d1	TGATGGAGGACTCGTATGCYTGT	0.125
	CocciEnv_R10d2	TGATGGARRACTCATATGCTTGT	0.125
	CocciEnv_R11d2	TGATAGAGAACTTGTATRCTTRT	0.125
	CocciEnv_R12d2	TGATGAAGAACTTRTATRCTTGT	0.125
	CocciEnv_R13d2	TGATRRAGGACTTGTATGCTTGT	0.125
	CocciEnv_R14	TGATGGAAAACTTGTATGCTTGT	0.125
	CocciEnv_R15d2	TGATGGAGGACTTGTAYAYTTGT	0.125
	CocciEnv_R16d2	TGATGGAGGACTTGTAYGCTTRT	0.125
	CocciEnv_R17d2	TGATGGAGGACTYATATGCTTRT	0.125
	CocciEnv_R18d2	GATGGAGGACTCGTWYGCTTGT	0.125
Taqman probe	CocciEnv_FMGB	6FAM-ACCCACATAGATTAGC-MGBNFQ	0.25

### ITS2 amplification and high-throughput amplicon sequencing

Nine samples comprising three low-positive sites (1, 3, 10), three moderate positive sites (8, 12, 15), and three high-positive sites (2, 4, 6) by *Coccidioides*-specific qPCR assay were subjected to Illumina-based amplicon sequencing with fungal-specific ribosomal DNA (rDNA) primers targeting the ITS2 region^54^. Briefly, a “two-step” amplification (as in ref. ^55^) was used to generate amplicon pools for sequencing. The first amplification round utilized the locus-specific PCR primers with a universal 5′ tail sequence (5′-CCTATGTGGAGAGCCAGTAAGCGATGCTATGGT-AACTTTYRRCAAYGGATCWCT-3′, 5′-GTCAACGCTCACTACTGCGATTACCCAAGTCAG-AGCCTCCGCTTATTGATATGCTTAART-3′). PCR was carried out in 25 μL reactions containing 1 × Phusion Green Hot Start II High-Fidelity PCR Master Mix (Thermo Fisher Scientific, Inc., Waltham, MA), an additional 1.5 mM MgCl_2_ (3.0 mM total), 200 nM each primer, and 2 μL each normalized DNA sample or 5 μL each dilute DNA sample (see above). Cycling conditions were 95 °C for 2 min, then 35 cycles of 95 °C for 30 s, 55 °C for 30 s, and 60 °C for 4 min. PCR products were checked on 1% agarose gel, purified by bead-prep, and diluted tenfold. Indexing and flowcell sequences were added in second round amplification using primers matching the universal tails at the 3′-end (5′-AATGATACGGCGACCACCGAGATCTACAC-NNNNNNNN-CCTATGTGGAGAGCCAGTAA-3′, 5′-CAAGCAGAAGACGGCATACGAGAT-NNNNNNNN-GTCAACGCTCACTACTGCGA-3′). Reaction conditions were the same as first round amplification, but containing only 100 nM each primer, 5 μL purified, diluted template, and cycling was carried out for only 15 cycles. PCR products were checked on 1% agarose gel, purified by bead-prep, quantified by PicoGreen fluorescence, and pooled together in equimolar quantities. The resulting pool was quantified by qPCR and inspected using a Bioanalyzer 2100 (Agilent Technologies, Santa Clara, CA) prior to sequencing on a MiSeq Desktop Sequencer (Illumina, Inc., San Diego, CA) in 2 × 300 bp mode. Custom sequencing primers were used which match the universal tail sequences from first round amplification (read 1: 5′-CCTATGTGGAGAGCCAGTAAGCGATGCTATGGT-3′; read 2: 5′- GTCAACGCTCACTACTGCGATTACCCAAGTCAG-3′; index 1: 5′-CTGACTTGGGTAATCGCAGTAGTGAGCGTTGAC-3′)

### Data analysis

Locus-specific primer sequences were removed from raw sequencing data prior to analysis using Cutadapt^56^ and paired end reads were merged with ea-utils^57^. Following inspection of merged reads in FastQC^58^ all sequences were trimmed uniformly to 300 nucleotides leaving an average *q*-score of q37. Data was demultiplexed in Quantitative Insights Into Microbial Ecology (QIIME)^59^ requiring 95% of each read to have a minimum *q*-score of 20, and allowing no exceptions (-*q* 19 -*r* 0 -*p* 0.95). All demulitplexed sequence data were deposited to GenBank under BioProject number PRJNA397457 with sample accessions SAMN07458979-SAMN07459014. Demultiplexed data was screened for chimeras with VSEARCH^60^ using the *usearch_ref* option against the UNITE-based fungal chimera dataset^61^, and subsequently screened for fungal ITS2 sequences with ITSx^62^. Sequences were dereplicated on the first 100 bases with the *prefix_suffix* OTU picker in QIIME. OTUs were defined de novo with Swarm^63^ at a resolution of 1, and taxonomy was assigned using BLAST^64^ against the UNITE database^65^. Reference sequences for *C. posadasii* and *C. immitis* were manually added to this database prior to taxonomic assignment. OTUs which represented <0.005% of the counts across the entire table were removed^66^. The OTU table was rarefied to the lowest depth sample (2593 reads) or normalized by CSS^67^ or DESeq2^68^ transformations. The rarefied table was used to explore differences in alpha-diversity using Chao1 estimator while all tables were used to explore differences in beta diversity or differential abundance of individual taxa using Bray-Curtis. Correlation between taxonomic identity and treatment groups was assessed with the Phi correlation from the R package ‘indicspecies’ at genus level^69^. Correlation between relative abundance of *Coccidioides* spp. ITS2 amplicons and qPCR Ct values was assessed using a two-tailed Spearman rank correlation test.

### Phylogenetic analysis

The 300 bp-amplicons representing OTUs specific to *Coccidioides* spp. were identified from each sampled location, and hereafter named denovo49 and denovo8395. Each of these OTUs was compared against the NCBI database (nr) by Nucleotide BLAST (BLASTn)^64^ to verify the identity of the OTU sequences, and to retrieve additional sequences for phylogenetic comparison. Retrieved ITS2 sequences for *C. posadasii* and *C. immitis* from isolates recovered from clinical or environmental samples were included for the phylogenetic comparisons. Sequences were aligned with MAFFT^70^ using the iterative refinement method (L-INS-I) and manually inspected. Sequences with low coverage compared to the query ITS2 fragment of *C. posadasii* were discarded and the remaining 72 aligned samples were subjected to phylogenetic analysis (see GENBANK accession numbers—Supplementary Table 1). A maximum likelihood (ML) tree was generated using the IQ-TREE software^71^. Models were calculated using the *-m TEST* function and branch support was tested using 1000 ultrafast bootstraps^72^. The bootstrap consensus tree was visualized with help of FigTree v1.4.3 (http://tree.bio.ed.ac.uk/software/figtree/).

## Results

### qPCR and mycological detection of *Coccidioides* spp. in Venezuelan soils

Different quantities of *Coccidioides* DNA were identified in the sampled areas of Venezeula (Table 3, Supplementary Fig. 1). Three sampled locations contained a high amount of *Coccidioides* DNA: Sites 2, 4, and 6. Those sites had real-time PCR amplification in all four DNA extractions from the site with low *C*_t_ values (<35), which indicates high concentrations of *Coccidioides* DNA in the sampled soils. Those samples were collected in Urumaco, Sucre, and Democracia. Sites 5, 9, 12, and 15 had at least two replicate DNA extractions positive, which we designated as moderate positive. Sites 1, 3, 7, 10, 11, 13, and 14 were designated as low positive, because we observed late amplification (*C*_t_ values >37) in only one of the four DNA extraction replicates (Table 3, Supplementary Fig. 1). No *Coccidioides* sp. growth was observed in any agar plates inoculated with soil or from tissues recovered from soil-infected BALB/c mice.

**Table 3:** Mean of *C*_t_ values obtained for the qPCR-based CocciEnv assay

Site	Replicate 1	Replicate 2	Replicate 3	Replicate 4	
1	n.d.	n.d.	39.170457	n.d.	Low positive
2	32.44438067	33.684223	31.253591	32.57464933	High positive
3	38.0875965	n.d.	n.d.	n.d.	Low positive
4	31.043169	29.483968	31.108808	32.5819565	High positive
5	n.d.	37.40752733	37.943037	n.d.	Moderate positive
6	32.039917	30.76345	31.93577333	31.11106433	High positive
7	n.d.	38.681686	n.d.	n.d.	Low positive
8	31.93436033	30.68055833	n.d.	n.d.	Moderate positive
9	40.3225925	n.d.	n.d.	36.743083	Moderate positive
10	n.d.	n.d.	n.d.	39.8178845	Low positive
11	n.d.	n.d.	n.d.	39.017355	Low positive
12	39.985367	n.d.	38.475866	36.31935467	Moderate positive
13	n.d.	37.238985	n.d.	n.d.	Low positive
14	39.1133525	n.d.	n.d.	n.d.	Low positive
15	36.5966605	33.26852667	n.d.	37.1464845	Moderate positive

DNA extractions were performed in quadruplicates for each site analyzed due to the likelihood of variable and potentially rare presence of *Coccidioides* DNA in the Lara and Falcon states of Venezuela. *C*_t_ values are averages of three replicate reactions for each DNA sample. n.d. not detected

The sites where samples 1–5 and 15 were collected are characterized by shrubby tropophilous deciduous, semi-deciduous, or deciduous basimontane tropophilous forests, and soils are predominantly clay where the samples had higher rates of positivity **(**Tables 1 and 3**)**. The locations 6–8 contain a spiny xerophytic bush-type vegetation and French-sandy soil. Sites 6 and 8 both had early *Coccidioides* DNA amplification *C*_t_ curves, reflecting higher amount of *Coccidioides* DNA. In contrast, the sites 9–11 from Urdaneta municipality are characterized by intense agricultural activity, clay-sandy soil type and part of the natural environment was disturbed by agricultural activity. Those sites had very low or negative detection for *Coccidioides*. The sites 12–14 present a thorny xeric shrublands vegetation-type and predominantly clay soil **(**Table 1**)**. This area has favorable phytophysiognomy conditions for *Coccidioides* development, but those samples had late or no real-time amplification of *Coccidioides* DNA. We identified plant species that are commonly distributed in arid and semi-arid areas in South America and may be associated with the presence of *Coccidioides* in the soil. Some particular plant species were found in highly positive soils: (1) Cactaceae family; *Opuntia wentiana, O. caracasana, O. caribaea, Melocactus sp., Cereus fricii, Cactus caesius* and (2) Fabaceae family*; Prosopis juliflora, Caesalpinia coriaria, Cercidium praecox, Acacia tortuosa, A. macracantha, Senna tora, Pseudopiptadenia pittieri***(**Supplementary Table 2**)**. Those species are commonly found in arid/semi-arid land ecosystems of the American continent and are adapted to similar extremotolerant (high pH/temperatures and limiting water) conditions as *Coccidioides immitis* and *C. posadasii*.

### Mycobiome sequencing

A total of 733,220 ITS amplicon sequences were retained following removal of low quality reads. After data processing, the 36 experimental samples (9 sites × 4 DNA replicates) yielded a median sequencing depth of 19,505 reads, ranging from 2593 to 50,351 reads. Sequences were classified into 351 distinct OTUs comprising 147 taxonomic assignments (Mean OTUs per taxonomic assignment = 2.38).

Two OTUs, herein named denovo49 and denovo8395, were found to represent *C. posadasii* in all sites. According to BLASTn analyses, sequences representing these OTUs each share 99% sequence identity with *C. posadasii* (Genbank accession number KR109218). The best BLAST hit of both OTUs corresponds to a sequence obtained from a *C. posadasii* isolate recovered from an Italian nun who visited Argentina for work (Supplementary Table 2). Although ITS2 sequences representing *C. posadasii* OTUs were observed in all nine sampled areas, the relative abundance of these OTUs varied according to the fungal load and was marginally correlated with *C*_t_ amplification curve of qPCR experiments (Spearman’s *R* = −0.661, *p* = 0.053). We detected a variation of 3806 × in number of *C. posadasii* amplicons comparing low-positive and high-positive sites that may reflect the amount of *Coccidioides* load, which would be associated with increased chances of pathogen exposure **(**Table 4, Supplementary Fig. 2**)**. For example, sites 2, 4, and 6 (highly positive) yield a higher number of *C. posadasii* amplicons compared to other low-positive sites **(**Table 4, Supplementary Fig. 2**)**. Both denovo49 and denovo8395 OTUs were identified in highly positive samples, but the absolute amounts varied according study sites. Sites 2 and 6 harbor predominantly OTU denovo49, whereas site 4 had an enrichment of OTU denovo8395 (Supplementary Fig. 2).

**Table 4:** Number of *C. posadasii* ITS2 amplicons sequenced for each of the four replicate DNA extractions representing each analyzed site in Lara and Falcon states of Venezuela

Site	Replicate 1	Replicate 2	Replicate 3	Replicate 4
1	1.1	1.2	1.3	1.4
denovo49	274	246	356	249
denovo8395	0	6	17	4
2	2.1	2.2	2.3	2.4
denovo49	423	235	211	611
denovo8395	7	13	43	0
3	3.1	3.2	3.3	3.4
denovo49	0	0	0	4
denovo8395	0	0	0	0
4	4.1	4.2	4.3	4.4
denovo49	9	290	26	2
denovo8395	455	3516	9	0
6	6.1	6.2	6.3	6.4
denovo49	209	450	4	88
denovo8395	0	145	0	0
8	8.1	8.2	8.3	8.4
denovo49	59	6	3	28
denovo8395	22	49	0	0
10	10.1	10.2	10.3	10.4
denovo49	1	0	0	2
denovo8395	0	0	0	0
12	12.1	12.2	12.3	12.4
denovo49	1	4	1	87
denovo8395	0	0	0	0
15	15.1	15.2	15.3	15.4
denovo49	7	2	3	1
denovo8395	0	0	0	0

**Fig. 1:**
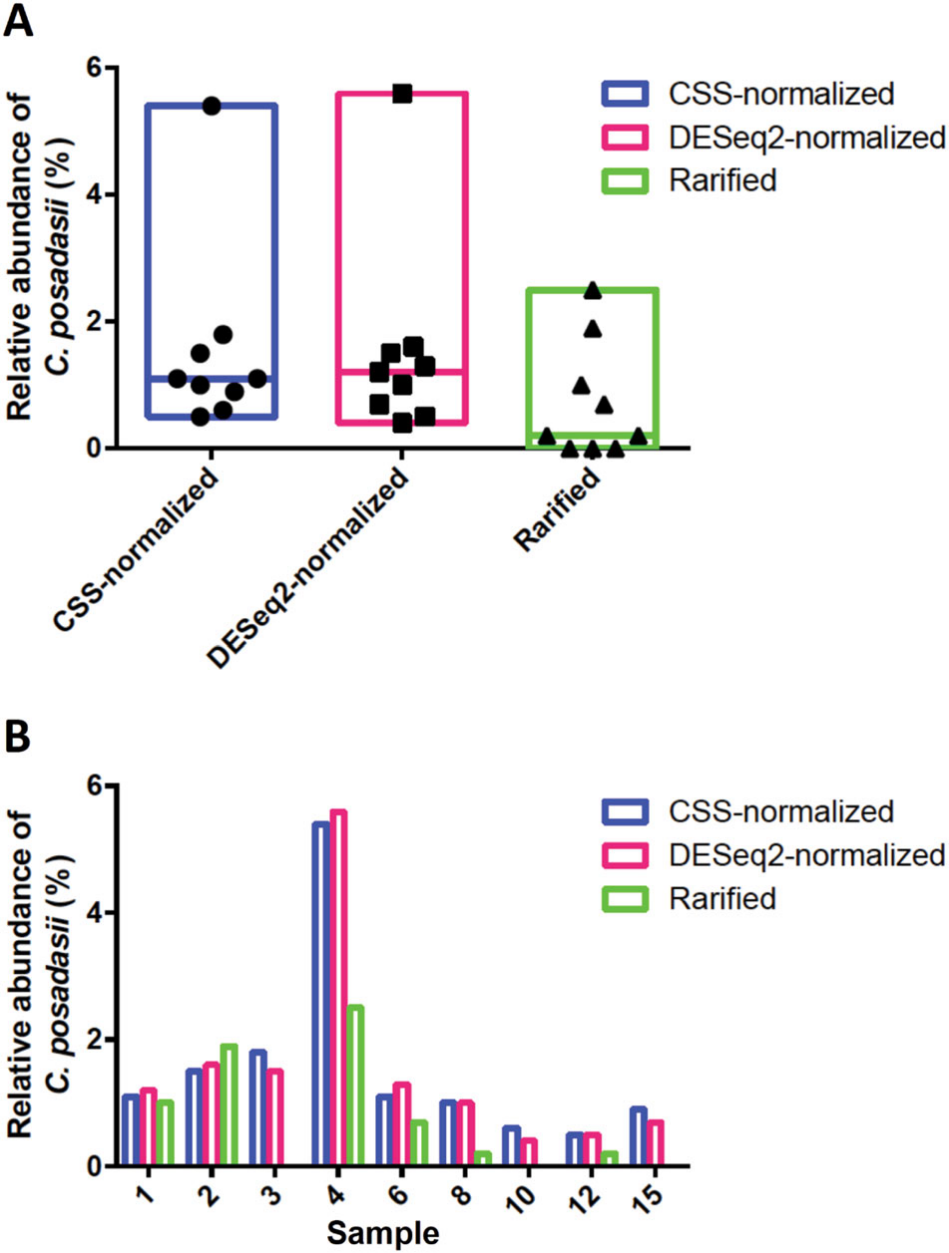
*C. posadasii* relative abundance in soil samples from Venezuela. **a** Overall relative abundance of *C. posadasii* comparing three different methods: rarefied to the lowest depth sample (Rarified) or normalized by CSS or DESeq2 transformations. **b** Site-specific relative abundance of *C. posadasii* comparing the three matrix normalization methods mentioned above

The percentage of *Coccidioides*-specific OTUs was as high as 5.6%, depending on the method used for differential abundance analysis implemented in QIIME (Fig. 1a). The *C. posadasii* abundance revealed in each of the nine sites between the three methods was plotted for each corresponding method. We observed consistent abundance of *C. posadasii* amplicons across sites, regardless of the OTU table used for analysis (CSS or DESeq2 normalization or rarefaction), though the rarified analysis provided a more conservative result (Fig. 1b). We analyzed the correlation in the increase of *C. posadasii* amplicons with the qPCR results, which showed that highly positive sites (2, 4, and 8) displayed an overall increased abundance of *C. posadasii* taxa compared to the low-positive sites (3, 10, 12). Moreover, the samples that yield low or no qPCR signal had zero or low levels of *C. posadasii* in rarified analysis (Fig. 1b). Surprisingly, Site 1, which was low positive for the qPCR experiment, actually showed a high number of *C. posadasii* amplicons, possibly due to an inhibitory component present in the soil DNA extraction that was removed during sample purification for ITS2 PCR and amplicon sequencing.

**Fig. 2:**
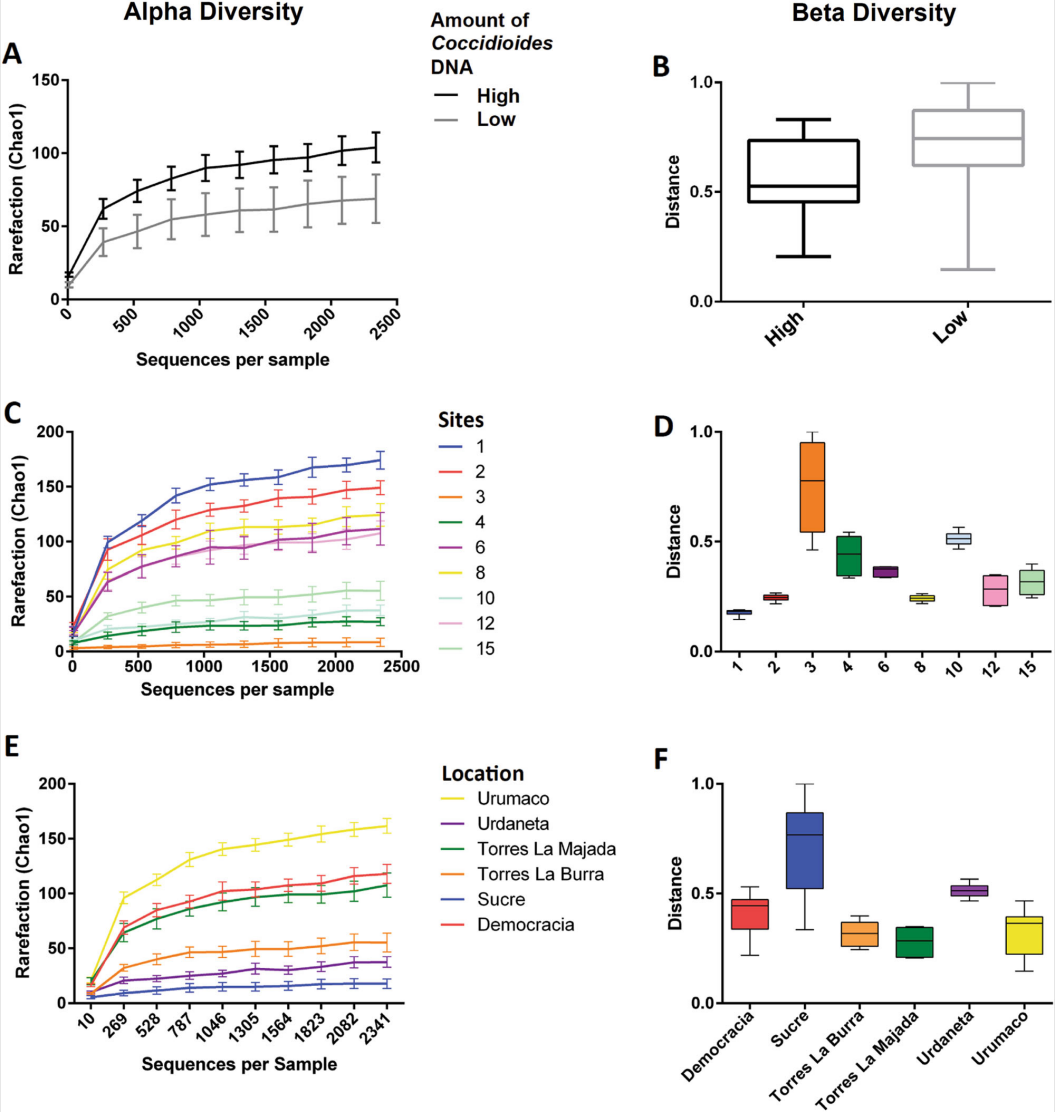
Alpha and Beta species diversity index calculated for the fungal communities observed in Lara and Falcon states of Venezuela. Species diversity indexes were calculated based on the amount of *C. posadasii* DNA in the soil (**a**, **b**), individual sites (**c**, **d**), and Venezuelan municipalities (**e**, **f**)

Community-level differences were assessed using alpha (Chao1) and beta (Bray-Curtis) diversity analysis in order to infer richness and distribution of the mycobiome in the sampled areas of Venezuela. Alpha and Beta diversity were calculated based on the rarefied OTU table for each site, or within municipalities, or based on the load of *C. posadasii* DNA detected by qPCR. The richness of samples was directly linked to the presence of *C*. *posadasii* in the soil. According to the rarefaction plots, the overall number of observed species was increased in the highly positive sites and decreased in the samples for which *C. posadasii* was rare or absent (high *C. posadasii* load 82.96 ± 35.35 vs. low *C. posadasii* load 53.59 ± 50.26, nonparametric *t*-stat = 1.996, *p* = 0.051 Fig. 2a, b). The number of observed species in sites 1 and 2 was highest when compared to site 3, which had the lowest mycobiome alpha-diversity (Fig. 2c, d). The evenness at high positive sites is lower, which indicated a more heterogeneous fungal community. The alpha and beta diversity index within municipalities of Venezuela (Falcon and Lara states) was higher in Urumaco (low-positive site) compared to Sucre that was shown to contain high amounts of *C. posadasii* DNA, but overall there was not a consistent pattern **(**Fig. 2e, f**)**.

We implemented a group significance analysis using the R package ‘‘indicspecies’’ at genus level in order to identify other fungal communities associated with the presence or scarcity/absence of *C. posadasii* in the soil. Sites which were determined as “highly positive” for *Coccidioides* by qPCR were correlated with seven additional taxa after FDR multiple correction as determined by Phi correlation (*p* < 0.05); Fungi sp., Chytridiomycetes sp., Chaetothyriales sp., Pleosporales sp., Dothioraceae sp., (unidentified taxa), *Trichoderma* sp., and *Ajellomyces* sp. The Dothideomycete (Ascomycota) fungal species *Camarosporium psoraleae* is present in sites (top ten species) 1, 2, 4, and 6 but was not statistically significant within the *C. posadasii*-positive group (data not shown). In addition, other Onygenalean species were found in possible association with *C. posadasii* including *Ajellomyces dermatitidis* and *Spiromastixa sexualis* found in sites 1, 2, 8, and 10; while *Gymnoascus* sp. 15PA08 occurred in sites 2, 10, and 12. Thus, these analyses suggest a composition of *C. posadasii*-associated taxa that may be correlated due to the presence of this pathogen in the arid/semi-arid environments, but additional investigation is required to define community associations.

### Phylogenetic distribution of *C. posadasii* OTU’s denovo49 and denovo8395

**Fig. 3:**
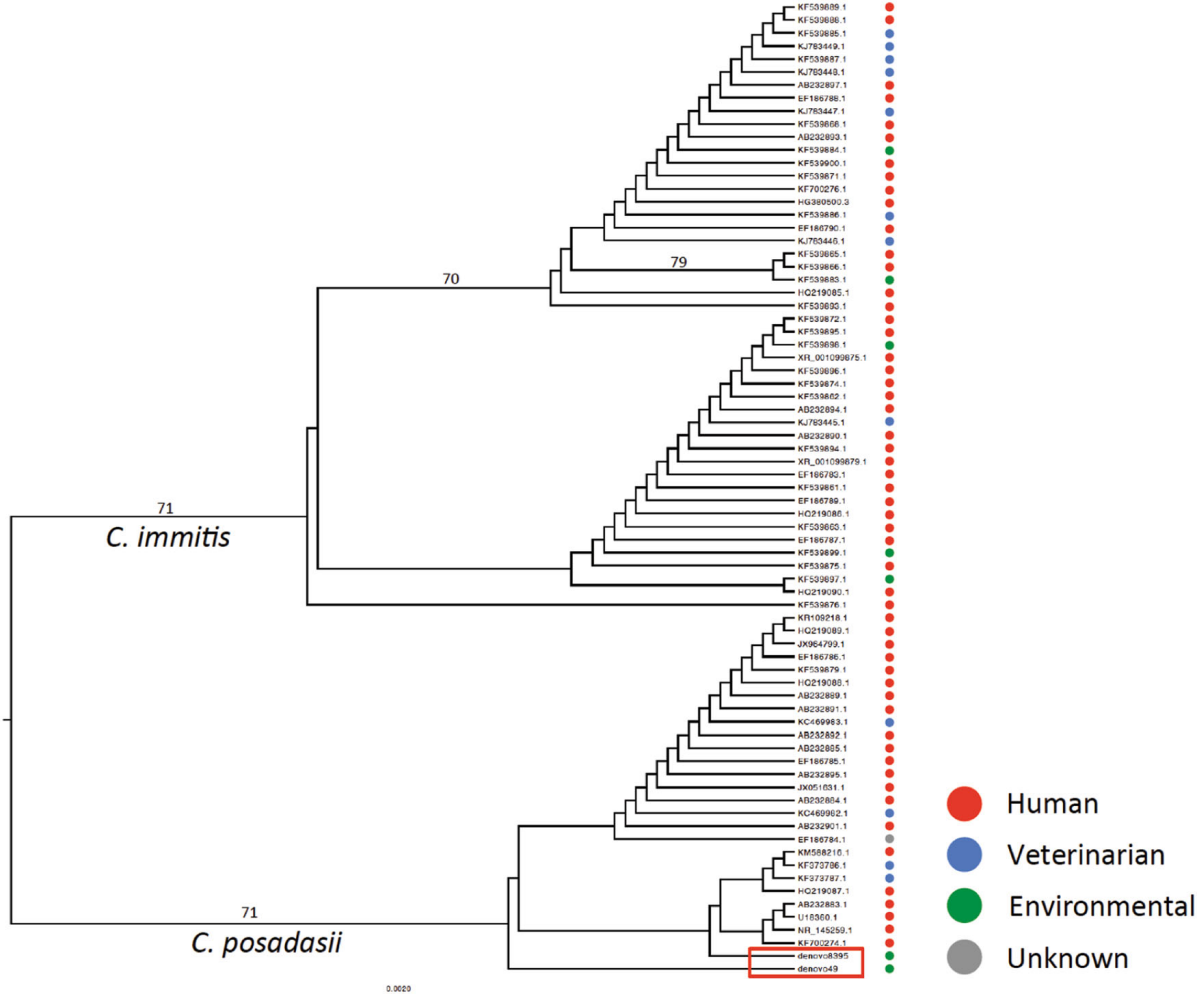
Maximum likelihood (ML) tree of the 300 bp fragment of ITS2 locus of environmental, veterinarian, and clinical samples of *Coccidioides immitis* and *C. posadasii*. Branch lengths are proportional to the number of genetic changes and bootstrap values >70% are displayed next to the branches

To understand the genetic origin of the *Coccidioides* taxa found in Venezuelan soil samples we compared the OTU’s denovo49 and denovo8395 with ITS2 sequences deposited at NCBI from clinical, veterinarian, and environmental isolates from *C. immitis* and *C. posadasii* (Supplementary Table 1). One-hundred ITS sequences from both *Coccidioides* species were retrieved from NCBI and aligned with the OTU’s denovo49 and denovo8395. Thirty NCBI sequences were excluded due low coverage in the alignment, and the remaining 72 sequences were used to construct a ML tree. As expected, both denovo49 and denovo8395 OTU’s clustered within *C. posadasii* and not *C. immitis* (Fig. 3). Interestingly, the OTUs denovo49 and denovo8395 were genetically distinct, which suggests that at least two *C. posadasii* genotypes may exist in Venezuela. We also compared the position of a clinical Venezuelan strain (Accession number KF539894), which clustered with *C. immitis* and not within *C. posadasii* (Fig. 3). As the majority of South American strains genotyped to date are *C. posadasii*, this unexpected observation could be the result of low levels of *C. immitis* in South America, patient travel history, or sample misidentification.

## Discussion

*C. immitis* is found in California, Washington (USA) and Northern Mexico; however, recent reports suggest the rare occurrence of this genotype in South America^21, 73^. The sister species, *C. posadasii* has a broader distribution, occurring in desert/semi-desert areas of Southwestern USA, Western Mexico, Central, and South America^21, 74^. We show that *C. posadasii* is endemic to municipalities of arid and semi-arid areas of Venezuela by detecting *Coccidioides posadasii*-specific DNA in all sampled areas (Fig. 1, Tables 1, 2, and 4). This geographic location where *C. posadasii* was identified is defined by the Paraguaná xeric scrubland^75^. These arid and semi-arid regions of Venezuela emerged during the Pliocene, and occupy two regions: the coastal plains of the Paraguaná peninsula and the depression valleys of Lara and Falcon^75^. These valleys are surrounded by the Andes and the Venezuelan Coastal Range and are considered an important extension of ancient Quaternary and recent sediments^75^. The annual precipitation varies from 350 mm in the semi-desert areas to 1000 mm in the areas of the Andean piedmont, and soil temperatures may reach 50 °C at ground level^76^. The Paraguaná peninsula lacks high flow perennial rivers, rather washes, and temporary streams are common geographical features. However, the depression valleys of Lara and Falcon do contain the Turbio, Mitare, and Tocuyo River systems that cross the Lara Falcón Dry Forest, and represent the main drainage area of the Paraguana xeric scrubland. Similarly, the endemic areas of coccidioidomycosis in Guatemala and Honduras are co-localized with Motagua and Comayagua valleys, respectively, which also are xeric shrubland biomes of Central America^41^. With respect to the phytophysiognomy, the Venezuelan coastal area is characterized by herbaceous-bushy vegetation, sand dunes, and saline depressions. In comparison, the Lara-Falcon depression is characterized by xerophyte plants, deciduous trees, and high concentrations of perennial trees that characterize the phytophysiognomies of the *Coccidioides*-positive sampled areas in this study (Table 1). We have identified a number of plant species from Fabaceae and Cactaceae plant families (Supplementary Table 2), which also are major constituents of the plant community in the Sonoran Desert and the Caatinga biome in Northeast Brazil where *C. posadasii* is endemic. According to Alarcón^75^ the unique biological community of the Lara-Falcon depression was influenced by high-speed northeast trade winds associated with the presence of high-salt concentration in the soil (especially calcite), and specific ecological adaptations are needed to facilitate plant, animal, and microorganism survival in these harsh environments.

Defining the key factors that explain presence (or absence) of *Coccidioides* in the environment remains an elusive problem. In fact, previous research suggests that the majority of soil samples tested are negative, and recovery rates in randomized collection studies are low^8, 11, 31, 37, 39, 77^. We showed that molecular methods based on real-time PCR and ITS2-amplicon sequencing are powerful tools to detect and indirectly quantify this pathogen in the environment and should be used for tracking this organism in the soil and inferring potential infection sites. We observed variation in the presence of *C. posadasii* depending on location, which suggests that the presence of this fungus varies, possibly due microenvironmental conditions. However, all attempts using both methodologies to recover the fungus from the soil were unsuccessful. Our molecular-based methods may overcome the limitations associated with isolation-based techniques that are laborious, and involve murine models and BSL-3 laboratory requirements for handling the fungi. Although the presence of DNA in the soil does not indicate fungal life stage or viability, it is a way to increase sampling to better understand the ecology and distribution of *Coccidioides* in the environment.

Previous examination of soil microbes cultured from *Coccidioides immitis* positive sites in California revealed antagonistic relationships between other organisms and *Coccidioides*^78, 79^. Direct plating from soil often results in overgrowth of *Coccidioides* colonies by other fungi^31, 34, 77^. *Coccidioides* also persists for many years in the same location^31, 77^. Herein, we showed that the increased presence of *C. posadasii* in the soil is statistically related to an increase of the overall number of fungi present at a given site, but an overall more heterogeneous fungal community **(**Fig. 2**)**. This suggests that there are important factors such as water availability, temperature and nutrition sources that may act as a selective pressure on fungal communities in semi-desert areas. In support of this hypothesis, we identified different specific fungal OTUs that are enriched in high loads of *C. posadasii* in the soil, suggesting that there are, in fact, specific fungal communities associated with the presence of *C. posadasii* in the soil. However, it is premature to broadly infer microbial entities related to the presence of *C. posadasii* since this species is distributed over different biomes. In addition, bacterial and viral communities were not explored in the present study, and may also play an important role in the distribution of *Coccidioides* in the environment.

We confirmed that *C. posadasii* is endemic to Venezuela, and we detected variation among the genotypes found in the soil compared to clinical samples in South America (Fig. 3). For example, the OTU denovo49 branched apart from the OTU denovo8395; this latter is more related to clinical *C. posadasii* genotypes. Unfortunately, our phylogenetic analysis is restricted to a 300-bp ITS2 fragment, which has low variation within species complexes. In order to prove true genotypic differences, isolation methods to obtain cultures are required (ongoing work) and the development of other amplicon targets is warranted. Recent population genetics studies revealed that there are significant genetic differences between environmental and clinical isolates recovered from Tucson, AZ, USA^23, 31^. Clinical isolates of *Coccidioides* species have an increase in proteases needed to degrade animal-derived material, and have experienced reduction of plant-degrading enzymes^24^. There is a possibility of genotypes in the environment which are adapted to alternative nutritional sources, such as insects or plants, and potentially be less pathogenic in a mammalian host.

Variation among locations where *Coccidioides* is found is evident, and this may select for different genotypes. For example, Tucson is located on the edge of the Sonoran Desert, and is defined as desert upland, whereas Yuma and Phoenix are in the lower elevation Sonoran Desert. In Arizona, the prevalence of *C. posadasii* appears to be associated with sandy and porous soil and rodent burrows^19, 31, 34, 35^. Variation in soil type and temperature, precipitation patterns, natural hosts, and vegetation will exert differential selection pressures on the fungus, which could explain the phylogenetic divergence between clinical and environmental isolates^11, 80^.

Using a combination of a targeted real-time PCR assay that amplifies a *Coccidioides*-specific target, and an amplicon sequencing approach, we were able to provide greater support of the ability to detect this fungal pathogen in the soil and monitor coccidioidomycosis outbreaks. The amplicon sequencing methodology can be scaled up for a diverse range of fungal pathogens, and bioinformatic workflows can be developed by utilizing specific databases such as the UNITE^81^ or ISHAM ITS databases (http://its.mycologylab.org/) so that both quantification and precise genotyping may be done in a single procedure using data from many different sequencing platforms. Additionally, by further characterizing the ITS2 sequences in a phylogenetic context, we were able to have a small window into the diversity of organisms that are present at a given site. This observation, coupled with a previous analysis, which compared clinical and environmental isolates using nine microsatellite loci, supports the hypothesis that there is greater diversity in the genus than has been identified to date^23, 31^. Indeed, this could in part explain a long-standing question in the field of coccidioidomycosis and other endemic mycosis research—why is there such variation in disease outcomes? At least 60% of coccidioidomycosis infections result in asymptomatic infection, and the same is generally observed in primary fungal pathogens. Could these infections be the result of exposure to cryptic low virulence genotypes that are found in the environment? The tools are now available for metagenome or direct amplicon sequencing of soils, which can help to address both fungal burden and genetic background of sources of coccidioidomycosis and other mycotic infections.

## Electronic supplementary material

Table S1(PDF 73 kb)

Table S2(PDF 61 kb)

Figure S1(DOCX 1122 kb)

Figure S2(DOCX 122 kb)
