# Supplementary material for: Detection of Coccidioides posadasii from xerophytic environments in Venezuela reveals risk of naturally acquired coccidioidomycosis infections
Source: Emerg Microbes Infect. 2018 Mar 29;7:46. doi: 10.1038/s41426-018-0049-6 (PMC5874253; doi:10.1038/s41426-018-0049-6)
Supplement: Supplementary file 1 — Table S1(PDF 73 kb) [file 41426_2018_49_MOESM1_ESM.pdf]

Table S1. Accession number, isolate and species identification, country and source of isolation for each taxa analyzed in the ITS2 phylogenetic tree in comparison with the soil OUT's identified in Venezuela.

**Genbank Accession number**	**Isolate**	**Species**	**Country**	**Location**	**Source**
KF539889	M4890	*C. immitis*	USA	Santa Barbara County	Human
KF539888	SL-2	*C. immitis*	USA	Monterey County Coast	Sea Lion
KF539885	SL-2 (clone)	*C. immitis*	USA	Monterey County Coast	Sea Lion
KJ783449	5447-09SP	*C. immitis*	USA	California	Sea Otter
KF539887	SL-2 (clone)	*C. immitis*	USA	Monterey County Coast	Sea Lion
KJ783448	5447-09LU	*C. immitis*	USA	California	Sea Otter
AB232897	IFM 50995	*C. immitis*	Japan	Osaka	Human
EF186788	CBS 113852	*C. immitis*	Mexico	North Mexico, Coahuila	Human
KJ783447	5359-08LN	*C. immitis*	USA	California	Sea Otter
KF539868	K-692	*C. immitis*	USA	Kern County, CA	Human
AB232893	IFM 46868	*C. immitis*	Japan	Kanagawa, Yokohama	Human
KF539884	Soil 3A	*C. immitis*	USA	San Benedito County, CA	Soil
KF539900	AD-92	*C. immitis*	USA	N.I.	Human
KF539871	K-692 (clone)	*C. immitis*	USA	Kern County, CA	Human
KF700276	CM7056	*C. immitis*	USA	California	Human
HG380500	FMR 12608	*C. immitis*	Turkey	N.I.	Human
KF539886	SL-2 (clone)	*C. immitis*	USA	Monterey County Coast	Sea Lion
EF186790	CBS 113857	*C. immitis*	USA	Monterey County, CA	Human
KJ783446	4359-08LN	*C. immitis*	USA	California	Sea Otter
KF539865	M2937	*C. immitis*	USA	Santa Barbara County, CA	Human
KF539866	M2937 (clone)	*C. immitis*	USA	Santa Barbara County, CA	Human
KF539883	Soil 3A (clone)	*C. immitis*	USA	San Benedito County, CA	Soil
HQ219085	CDC_B727	*C. immitis*	Argentina	N.I.	Human
KF539893	M4890 (clone)	*C. immitis*	USA	Santa Barbara County	Human
KF539872.1	K-727	*C. immitis*	USA	Kern County, CA	Human
KF539895	Venezuela	*C. immitis*	Venezuela	San Miguel, Lara state	Human
KF539898	Whiskey Hill	*C. immitis*	USA	Colusa County, CA	Soil
XR_001099875	RS	*C. immitis*	USA		Human

KF539896	Venezuela (clone)	*C. immitis*	Venezuela	San Miguel, Lara state	Human
KF539874	M3429	*C. immitis*	USA	Santa Barbara County	Human
KF539862	95	*C. immitis*	USA	San Diego, CA	Human
AB232894	IFM 50992	*C. immitis*	USA	NI	Human
KJ783445	4275-04SP	*C. immitis*	USA	California	Sea Otter
AB232890	IFM 45815	*C. immitis*	USA	NI	Human
KF539894	Venezuela (clone)	*C. immitis*	Venezuela	San Miguel, Lara state	Human
XR_001099879	RS	*C. immitis*	USA		Human
EF186783	CBS 166.51	*C. immitis*	USA	NI	Human
KF539861	46	*C. immitis*	USA	NI	Human
EF186789	CBS 113856	*C. immitis*	Mexico	South Mexico, Michoacan	Human
HQ219086	RMSCC2395	*C. immitis*	USA	California	Human
KF539862	95 (clone)	*C. immitis*	USA	San Diego, CA	Human
EF186787	CBS 113851	*C. immitis*	USA	San Diego, CA	Human
KF539899	Whiskey Hill (clone)	*C. immitis*	USA	Colusa County, CA	Soil
KF539875	M3429	*C. immitis*	USA	Santa Barbara County	Human
KF539897	Whiskey Hill (clone)	*C. immitis*	USA	Colusa County, CA	Soil
HQ219090	RMSCC2009	*C. immitis*	USA	San Joaquin Valley, CA	Human
KF539876	M3429	*C. immitis*	USA	Santa Barbara County	Human
KR109218	RKI05-0686	*C. posadasii*	Italy (Argentina)	N.I.	Human
HQ219089	RMSCC1036	*C. posadasii*	USA	Tucson, AZ	Human
JX964799	NCCPF 230018	*C. posadasii*	India	N.I.	Human
EF186786	CBS 113846	*C. posadasii*	USA	Tucson, AZ	Human
KF539879	Silveira	*C. posadasii*	USA	San Joaquin Valley, CA	Human
HQ219088	RMSCC2343	*C. posadasii*	Mexico	Nuevo Leon	Human
AB232889	IFM 45813	*C. posadasii*	USA	New York, NY	Human
AB232891	IFM 45816	*C. posadasii*	USA	N.I.	Human
KC469983	IDR1200041772	*C. posadasii*	USA	New York, NY	Rhesus Monkey kidney cells
AB232892	IFM 45817	*C. posadasii*	USA	N.I.	Human
AB232885	IFM 45809 (Silveira)	*C. posadasii*	USA	San Joaquin Valley, CA	Human

EF186785	CBS 113843	*C. posadasii*	USA	San Antonio, TX	Human
AB232895	IFM 50993	*C. posadasii*	Japan	Ishikawa, Kanazawa	Human
JX051631	CEMM 05-4-10	*C. posadasii*	Brazil	Santa Quitéria, Ceará	Human
AB232884	IFM 4945	*C. posadasii*	Japan	Tokyo	Human
KC469982	IDR1200041771	*C. posadasii*	USA	New York, NY	Rhesus Monkey kidney cells
AB232901	IFM 54196	*C. posadasii*	Japan	Chiba	Human
EF186784	CBS 196.34	*C. posadasii*	N.I.	N.I.	N.I.
KM588216	Silveira	*C. posadasii*	USA	San Joaquin Valley, CA	Human
KF373786	S1203408.1	*C. posadasii*	USA	Somis, Ventura County, CA	Alpaca
KF373787	S1203408.2	*C. posadasii*	USA	Somis, Ventura County, CA	Alpaca
HQ219087	RMSCC2233	*C. posadasii*	USA	San Antonio, Texas	Human
AB232883	IFM 4935	*C. posadasii*	Japan	Aichi, Nagoya	Human
U18360	ATCC 28868	*C. posadasii*	USA	California	Human
NR_145259	ATCC 28868 (clone)	*C. posadasii*	USA	California	Human
