# Supplementary material for: Detection of Coccidioides posadasii from xerophytic environments in Venezuela reveals risk of naturally acquired coccidioidomycosis infections
Source: Emerg Microbes Infect. 2018 Mar 29;7:46. doi: 10.1038/s41426-018-0049-6 (PMC5874253; doi:10.1038/s41426-018-0049-6)
Supplement: Supplementary file 2 — Table S2(PDF 61 kb) [file 41426_2018_49_MOESM2_ESM.pdf]

Table S2. Plant species and vegetation type identified in the sampled areas in the Lara and Falcon states of Venezuela

Sites – Vegetation type	Plant species
1 and 2 - *Urumaco/San José de Bruzual* - Shurbs tropophilous deciduous and semi-deciduous	· *Opuntia wentiana*<br>· *O. caracasana*<br>· *O. caribaea*<br>· *Prosopis juliflora*<br>· *Crysophyllum sp.*<br>· *Cereus fricii*<br>· *Acacia macracantha*<br>· *Caesalpinia coriaria*<br>· *Melocactus sp.*<br>· *Tabebuia serratifolia*
3, 4 and 5 - *Sucre (Pedregal)* - Tropophilous forest basimontane	· *Opuntia caribaea*<br>· *O. wentiana*<br>· *O. caracasana*<br>· *Prosopis juliflora*<br>· *Chrysophyllum sp.*<br>· *Acacia macracantha*<br>· *Acacia tortuosa*<br>· *Bulnesia arborea*<br>· *Coelogyne odoratissima*<br>· *Tabebuia serratifolia*
6, 7 and 8 - *Democracia (Pecaya)* - Spiny xerophytic bushes	· *Opuntia caribaea*<br>· *O. caracasana*<br>· *Melocactus sp.*<br>· *Cercidium praecox*<br>· *Acacia tortuosa*

- *Bulnesia arborea*
- *Prosopis juliflora*
- *Cereus fricii*
- *Tabebuia serratifolia*

9, 10 and 11 - *Urdaneta (Siquisique)*

- *Opuntia caracasana*
- *O. wentiana*
- *O. caribaeae,*
- *Prosopis juliflora*
- *Cercidium praecox*
- *Rhodocactus guamacho*

12, 13 and 14 - *Torres (La Majada)*

- *Cactus caesius*
- *Opuntia caracasana*
- *Opuntia wentiana*
- *Prosopis juliflora*
- *Tabebuia sp.*
- *Alternanthera ficoidea.*

15 - *Torres (La Burra)*

- *Senna tora*
- *Caesalpinia coriaria*
- *Protium heptaphyllum*
- *Pseudopiptadenia pittieri*
- *Parinari pachyphylla*
- *Abutilon piramide*
- *Digitaria insularis*
