# Supplementary figures and images for: Detection of Coccidioides posadasii from xerophytic environments in Venezuela reveals risk of naturally acquired coccidioidomycosis infections
Source: Emerg Microbes Infect. 2018 Mar 29;7:46. doi: 10.1038/s41426-018-0049-6 (PMC5874253; doi:10.1038/s41426-018-0049-6)

**Fig. S1 –** Sites of soil sampling in semi-arid municipalities of Falcon and Lara states of Venezuela.


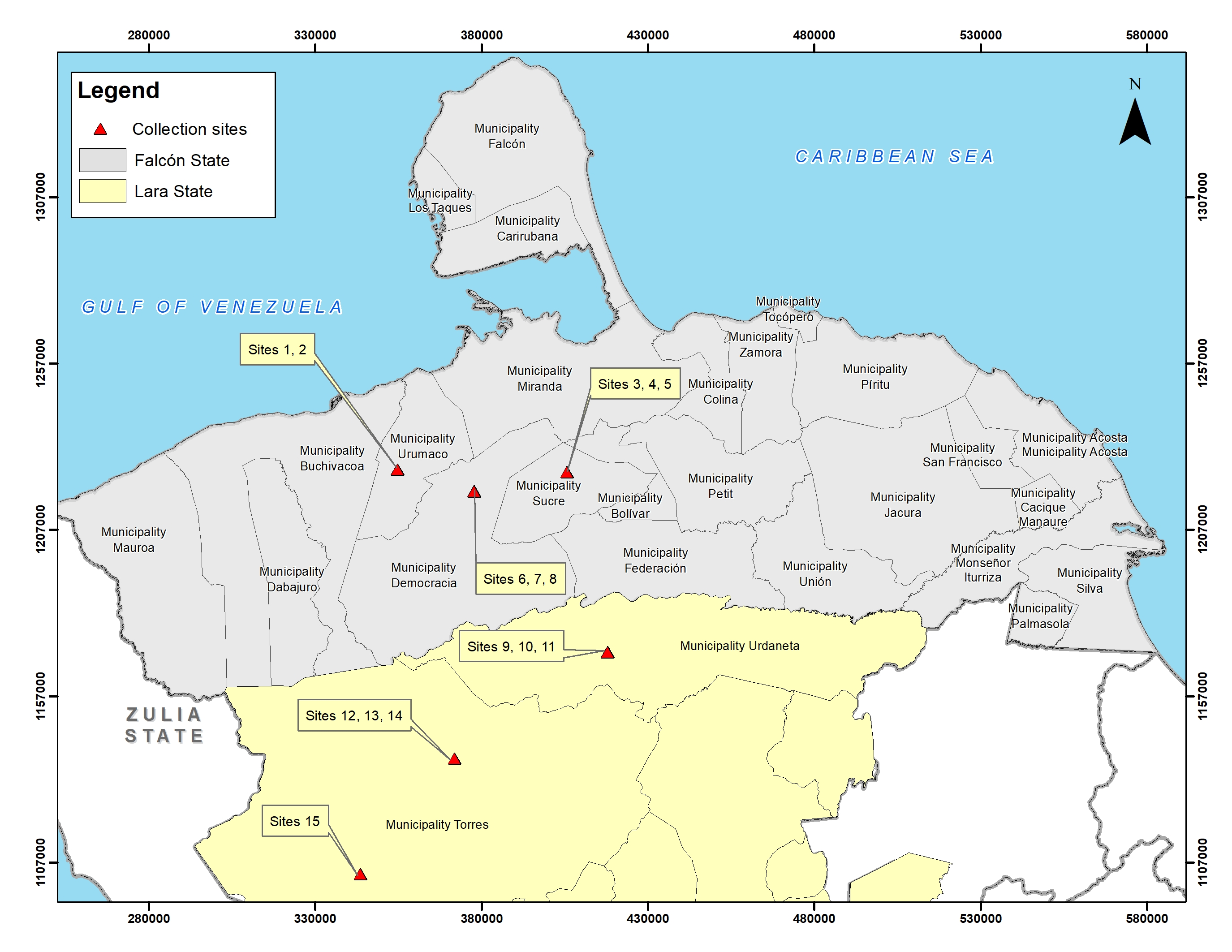

Supplement: Supplementary file 3 — Figure S1(DOCX 1122 kb) [file 41426_2018_49_MOESM3_ESM.docx]
