# Supplementary material for: Detection of Coccidioides posadasii from xerophytic environments in Venezuela reveals risk of naturally acquired coccidioidomycosis infections
Source: Emerg Microbes Infect. 2018 Mar 29;7:46. doi: 10.1038/s41426-018-0049-6 (PMC5874253; doi:10.1038/s41426-018-0049-6)

**Figure S2 -** Number of *C. posadasii* ITS2 paired-reads representing OTUs denovo49 and denovo8395 obtained for each of quadruplicates that corresponds a single location in semi-arid areas of Falcon and Lara states of Venezuela.


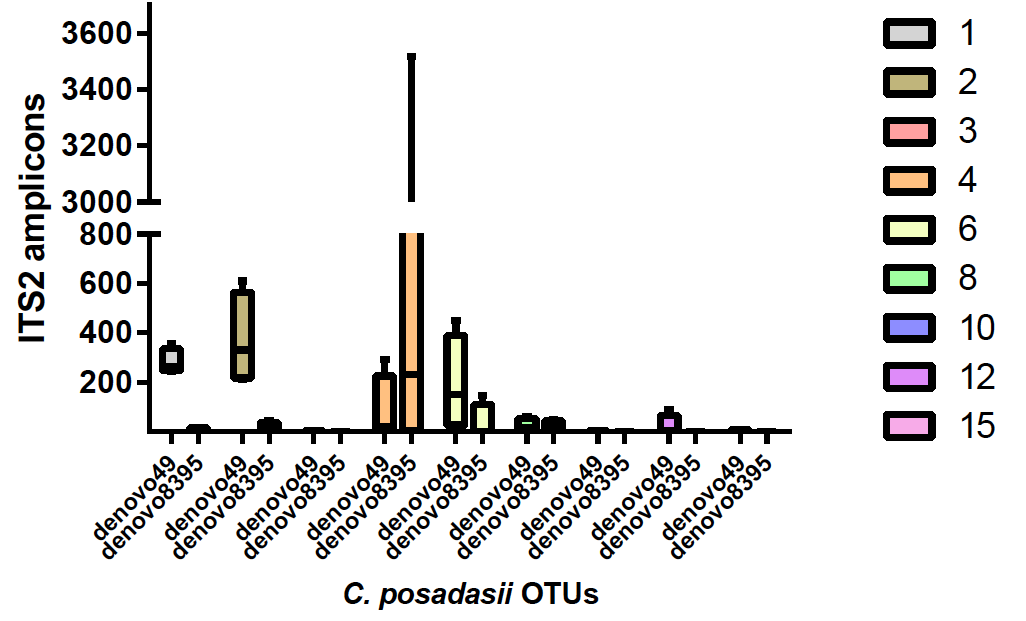

Supplement: Supplementary file 4 — Figure S2(DOCX 122 kb) [file 41426_2018_49_MOESM4_ESM.docx]
